# Design of a prospective clinical study on the agreement between the Continuous GlucoseMonitor, a novel device for CONTinuous ASSessment of blood GLUcose levels, and the RAPIDLab® 1265 blood gas analyser: The CONTASSGLU study

**DOI:** 10.1186/1471-2253-12-24

**Published:** 2012-09-22

**Authors:** Johannes B Zimmermann, Monika Lehmann, Stefan Hofer, Johannes Hüsing, Catharina Alles, Jens Werner, Jürgen Stiller, Wolfgang Künnecke, Steffen Luntz, Johann Motsch, Markus A Weigand

**Affiliations:** 1 Department of Anaesthesiology, University Hospital Heidelberg, Im Neuenheimer Feld 110, 69120, Heidelberg, Germany; 2 Coordination Centre for Clinical Trials, Building 4410, Voßstraße 2, 69115, Heidelberg, Germany; 3 Department of General-, Visceral-, and Transplantation Surgery, University Hospital Heidelberg, Im Neuenheimer Feld 110, 69120, Heidelberg, Germany; 4 Mechatronic AG, Wittichstraße 2, 64295, Darmstadt, Germany; 5 TRACE Analytics GmbH, Richard-Wagner-Straße 1-2, 38106, Braunschweig, Germany; 6 Department of Anaesthesiology and Operative Intensive Care, University Hospital Giessen and Marburg GmbH, Rudolf-Buchheim-Str. 7, 35392, Giessen, Germany

**Keywords:** Agreement, Continuous, Monitor* (monitor~s~ing), Blood glucose [MeSH], Lactate [MeSH], Insulin [MeSH], Potassium [MeSH], Perioperative care [MeSH], Critical care [MeSH], Intensive care

## Abstract

**Background:**

Although a device is needed to continuously measure blood glucose levels within an intensive care setting, and several large-scale prospective studies have shown that patients might benefit from intensive insulin, potassium, or glucose therapy during intensive care, no devices are currently available to continuously assess blood glucose levels in critically ill patients. We conceived the study described here to evaluate the clinical use of the Continuous Glucose Monitor (CGM) performed via a central vein, and to determine the impact of phenomena, such as drift and shift, on the agreement between the CGM and a RAPIDLab® 1265 blood gas analyser (BGA).

**Methods/design:**

In the CONTinuous ASSessment of blood GLUcose (CONTASSGLU) study, up to 130 patients under intensive care will be fitted with the CGM, an ex vivo device that continuously measures blood glucose and lactate levels. Readings from the device taken 8 h after initial placement and calibration will be compared with values measured by a BGA. For this study, we chose the BGA as it is an established standard point-of-care device, instead of the devices used in certified central laboratories. Nevertheless, we will also independently compare the results from the point-of-care BGA with those determined by a central laboratory-based device. Blood samples will be collected from each patient from the same site in which the CGM will measure blood glucose. Consequently, each participant will serve as their own control, and no randomisation is necessary. The 95% limits of agreement and the corresponding confidence intervals will be calculated and compared with a prespecified clinically acceptable relative difference of 20%.

**Discussion:**

Several attempts have been made to develop a device to continuously measure blood glucose levels within an intensive care setting or to use the devices that were originally designed for diabetes management, as several of these devices are already available. However, none of these devices were successful in intensive care settings. CONTASSGLU may well bridge this gap by confirming the ability of the CGM to continuously measure blood glucose levels in intensive care settings.

**Trial registration:**

ClinicalTrials.gov NCT01580176

## Background

TRACE Analytics GmbH (Braunschweig, Germany) and Mechatronic AG (Darmstadt, Germany), in a joint effort with the University Hospital Heidelberg (Germany), recently developed the Continuous GlucoseMonitor (CGM), an *ex vivo* device that continuously measures blood glucose and lactate levels. The CGM assesses blood glucose levels from a microdialysate of the circulating blood by an amperometric technique. It consists of a control unit and a sensor system with a sampling device.

Critically ill patients under intensive care, including patients with severe sepsis/septic shock, may have severe microcirculatory disturbances. These disturbances may cause tissue hypoxia [1]. Critically ill patients may also have metabolic disturbances, including hyperglycaemia or insulin resistance, even if they did not previously have diabetes [2,3]. Another common metabolic disturbance is acidosis, which may severely impair endothelial function [4]. Tissue hypoxia and respiratory acidosis may worsen this condition. Additionally, insulin resistance or insulin deficiency may induce metabolic acidosis [5]. If endothelial function to retain blood plasma is lost [6] fluid loss from the circulation may further diminish capillary blood flow [1]. The microcirculatory disturbances may maintain themselves and ultimately lead to death [7].

The sensor technology (i.e., the control unit and the sensor system) was confirmed to be functional in preliminary laboratory and preclinical tests via peripheral intravenous access to the vascular bed. However, it is not known whether these promising results also apply to central venous access.

Excess fluid loss may cause severe interstitial oedema [6], characterised by swelling of the face, body, and extremities. Oedema may also render peripheral intravenous access technically impossible for patients under intensive care, necessitating an alternative route.

### Objectives

The objective of the CONTinuous ASSessment of blood GLUcose (CONTASSGLU) study is to evaluate the clinical use of the CGM performed via a central vein, and to determine the impact of phenomena, such as drift and shift, on the agreement between the CGM and a RAPIDLab® 1265 blood gas analyser (BGA). To fulfil this objective, up to 130 patients under intensive care will be fitted with the CGM. Readings from the device taken 8 h after initial placement and calibration will be compared with values measured by a BGA.

## Methods/design

### Study design

CONTASSGLU is a prospective clinical study that is designed to assess the level of agreement between two devices used to measure blood glucose. Readings from the CGM will be compared with values measured by a BGA, an established device in clinical care. Blood samples used for the BGA measurement will be collected from each patient from the same site and at the same time at which the CGM will measure blood glucose. Consequently, each participant will serve as their own control, and no randomisation is necessary.

### Participants

Considering that patients with severe sepsis/septic shock cannot give written informed consent, and we could not justify asking the patient’s next of kin for written consent, we did not use this population of patients. Instead, we plan to enrol patients who are scheduled for major abdominal surgery, predominantly pancreatic surgery (pancreatectomy), for any reason and who are expected to be under intensive care for ≥ 8 h after surgery.

We will primarily focus on patients undergoing pancreatectomy for any reason (total or subtotal/distal pancreatectomy) to assess the level of agreement between the two devices over a wider range of blood glucose levels than would be possible in other groups of patients. The pancreatic islets of Langerhans provide critical control of blood glucose homeostasis. Consequently, blood glucose levels are expected to reach pathological values (i.e., dysglycaemia) immediately after pancreatectomy. Because the islets of Langerhans are mainly located in the pancreatic tail [8], patients undergoing total pancreatectomy or subtotal pancreatectomy (i.e. distal pancreatectomy) will be considered for admission to the study. Table 1 provides a comprehensive list of the eligibility criteria to be applied in this study.

**Table 1:** Eligibility criteria

**Patients meeting all of the following criteria will be considered for admission to the study:**
· Undergoing major abdominal surgery, predominantly pancreatic surgery (pancreatectomy), for any reason
· Require careful postoperative monitoring of blood glucose levels
· Expected to be under intensive care for ≥ 8 h after surgery
· Insertion of a multi-lumen central venous catheter for medical reasons (e.g., anaesthesia and operative purposes)
· Aged 18–80 years
· Able to understand the design and possible consequences of the clinical study
In addition, all patients must meet the following criteria, or will not be included in the study:
· Successful central venous cannulisations with a multi-lumen catheter
· One lumen is not needed for medical purposes, such as perioperative fluid therapy or medication administration
Patients fulfilling any of the following criteria will be excluded from the study:
· History of thrombosis, embolism, or vascular obliteration
· Bleeding disorders (e.g., thrombocytosis)
· Acute or chronic cardiac failure
· Acute or chronic kidney failure (i.e., patients on renal replacement therapy)
· Evidence of postoperative hyperhydration (pulmonary vascular congestion)
· Acquired immune deficiency syndrome
· Receiving immunosuppressive therapy
· Signs or symptoms of acute or chronic infection
· No perioperative antibiotic prophylaxis (with mezlocillin 4 g and metronidazole 500 mg, for example)
· No perioperative prophylaxis to prevent venous thromboembolism (e.g., unfractionated or fractioned/low-molecular weight heparin)
· The amount of flushing medium administered is > 500 mL
· Pregnancy or lactation
· Participation in other clinical studies and observation period of competing studies, respectively

No patient will be allowed to enrol in this study more than once.

### Study settings

The Department of Anaesthesiology, at University Hospital Heidelberg, Heidelberg, Germany will be responsible for recruiting, treating, and following all patients in this study. The department will be monitored by the Coordination Centre for Clinical Trials, which will be responsible for study management, medical device safety, data management, and statistical analyses.

### Outcome measures and endpoints

The CGM is specifically designed for use in intensive care medicine. However, for it to be successfully implemented in clinical use and to confirm the advantages of continuous assessment of blood glucose levels in this setting, the clinical workflow must be accounted for. At the University Hospital Heidelberg (Interdisciplinary Operative Intensive Care Unit), blood samples are collected for point-of-care blood gas analyses using a BGA, and blood glucose levels are assessed as a by-product every hour. Nurses change shift every 8 h.

In this setting, the BGA is established as the standard device, and is used instead of the larger devices used in certified central laboratories. Nevertheless, we will also independently compare the results from the point-of-care BGA with those determined by a central laboratory-based device. The level of agreement between values measured by the CGM and a BGA will be assessed ≥ 8 h after the initial placement and calibration of the CGM.

### Primary outcome measure

The primary outcome measure, referred to as the agreement at point of care, is defined as the level of agreement in blood glucose levels determined by the CGM and a BGA, assessed 8 h after the initial placement and calibration of the CGM.

The agreement for the *i*th patient will be quantified as the relative difference $d_{i}$ between readings ($y_{\mathrm{gi}}$) and measurement results ($y_{\mathrm{ri}}$), calculated as $d_{i}=\frac{2\left(y_{\mathrm{gi}}-y_{\mathrm{ri}}\right)}{y_{\mathrm{gi}}+y_{\mathrm{ri}}}$. Using the approach suggested by Bland and Altman [9,10] the limits of this agreement are set to $\bar{d}\pm 2s_{d}$, where $\bar{d}$ and $s_{d}$ are the empirical mean and the standard deviation of *d*_*i*_.

According to the guidelines for quality assurance in quantitative laboratory tests issued by the German Medical Association (GMA) [11,12], the measurement results from any device should not differ from an established standard by more than 15%. In this setting, however, the standard is not an unknown clinical value measured with a standard device but instead a prepared sample for which accuracy was achieved by titration or other methods. Unlike this method, the test–retest variance of the standard device contributes to the total error in our study. Moreover, the asymmetrical measure $\frac{1}{y_{0}}\sqrt{\frac{1}{n}{\left(y_{i}-\;y_{0}\right)}^{2}}$ is smaller than the measure $\left|\bar{d}\right|\pm 2s$ taken from the symmetrical limits of agreement under any but highly unlikely conditions. Thus $\bar{d}\pm 2s_{d}$ has been set to 20%, which is more conservative than the requirements imposed by the GMA. Therefore, the agreement at point-of-care is considered sufficient if the limits of agreement of the relative difference and their 95% confidence intervals (CIs) are within 20%.

### Secondary outcome measures and endpoints

Secondary outcomes include the following:

• Deteriorations in agreement between the CGM and BGA values measured in the short (1 and 2 h) and medium term (3–8 h) after initial placement and calibration of the CGM

• Agreement beyond point of care, defined as the agreement in blood glucose levels determined by the CGM and a BGA, recorded at 9 and 10 h after the initial placement and calibration of the CGM

• Long term deterioration in agreement measured at 16 and 24 h after initial placement and calibration of the CGM

• Rate of unwanted effects and events defined in DIN EN ISO 14155 2012–01 [Clinical investigation of medical devices for human subjects – Good clinical practice (ISO 14155:2011 + Cor. 1:2011)].

### Sample size

The agreement between the two devices will be quantified using the approach suggested by Bland and Altman, in which the 95% limits of agreement with 95% CIs will be compared with a prespecified, clinically acceptable relative difference [9] of 20%. The standard error of the 95% limits of agreement is approximately $\mathrm{se}\approx \sqrt{\frac{3s_{d}^{2}}{n}}$, where $s_{d}$ is the standard deviation of the differences between the two devices and n is the sample size (number of paired values the differences are calculated from). The sample size is dependent on the intended width of the CIs. Bland and Altman recommend a sample size of 100, which gives a 95% CI of about ± 0.34$s_{d}$, or about one-sixth of the width of the actual agreement. The power to reject the hypothesis that the prespecified limits of agreement of ± 20% are violated is > 80% if $\frac{\delta +\;2\sigma -\;\lambda }{\sqrt{\frac{3\sigma^{2}}{n}}}<-\;2.486$, where λ is the 20% limit and *δ* and $\sigma^{2}$ represent the expected value and variance of *d*, respectively. This inequality may be approximated by δ+2.5σ<λ indicating that the power is sufficient with *δ* = 5% and *σ* = 6%, for example. To fulfil the sample size recommended by Bland and Altman (i.e., ≥ 100 pairs of values), we plan to enrol up to 130 patients in this study.

### Feasibility

At University Hospital Heidelberg (Department of General, Visceral, and Transplant Surgery) 146 pancreatectomies are done every year, of which 48 and 98 are total and subtotal/distal pancreatectomies, respectively. These values were determined in 2009; values in 2010 have yet to be determined. DISPACT (ISRCTN18452029) [13,14], a trial examining techniques for closure of the remnant pancreas in distal pancreatectomy applied similar eligibility criteria, and confirmed that six patients could be enrolled every month. Therefore, the target sample size is achievable. Accounting for a slow starting period, recruitment is expected to take about 8 months.

### Interim analyses and stopping guidelines

Once 20 patients have been included, an interim analysis will be carried out with the possibility to stop the study for futility. This interim analysis is based on the conditional power approach [15]. Under the assumption of sufficient power (i.e., the probability that the alternative hypothesis is true, by a sufficient margin from the null hypothesis), the margin for the distribution of the test statistic $Z=\frac{\left|\bar{d}\right|+\;2s_{d}-\lambda }{\sqrt{3/ns_{d}^{2}}}$ is $\theta =u_{1-\alpha }+u_{1-\beta }$, where *u*_*l*_ is the *l*-quantile of the standard normal distribution. The interim test statistic is calculated as $Z_{1}=\frac{\left|\bar{d}\right|+\;2s_{d_{1}}-\lambda }{\sqrt{3/n_{1}s_{d}^{2}}}$, where *λ* = 20% is the 20% limit, and *Z*_1_, *d*_1_, and *n*_1_are the respective values after enrolling the first 20 patients. Given $\theta =u_{1-\alpha }+u_{1-\beta }$, the conditional power to successfully reject the null hypothesis is below 30% if *Z*_1_ > 0.813 for *n*_1_ = 20. If the alternative hypothesis is true and the true power is 80%, the probability of obtaining this result is < 0.05%, resulting in a nominal power of 79.95%. The interim analysis will safeguard against the situation where the level of agreement is too low for the study to justifiably continue.

### Sampling method

The CGM assesses blood glucose levels from a microdialysate of the circulating blood by an amperometric technique. In brief, acetated Ringer’s solution (perfusate) is delivered through a microdialysis probe. Solute exchange occurs in both directions across the semipermeable membrane of the microdialysis probe and enriches the perfusate by a fraction of the blood glucose level. This fraction is referred to as the relative recovery. Knowledge of the relative recovery rate is essential to determine solute levels [16]. The CGM is calibrated before implantation and is immediately referenced. Patency of access to the vascular bed is maintained by programmed infusion of normal saline solution supplemented with 1,000 IU/L heparin (flushing medium).

At the University Hospital Heidelberg (Interdisciplinary Operative Intensive Care Unit) point-of-care blood gas analyses are done using a RAPIDLab® 1265 blood gas analyser (BGA; Siemens Healthcare Diagnostics GmbH, Bad Nauheim, Germany). The BGA assesses blood glucose levels from lithium heparinised whole blood, also by an amperometric technique.

In brief, the amperometric technique involves the conversion of glucose and oxygen in water to gluconolactone and hydrogen peroxide, in a reaction catalysed by glucose oxidase. The concentration of glucose in the sample is determined indirectly as the amount of hydrogen peroxide yielded by the reaction. Hydrogen peroxide is detected on the surface of a platinum electrode upon oxidation resulting in a change of the electrochemical potential [17].

At the University Hospital Heidelberg (Central Laboratory), an ADVIA® 2400 Chemistry System (Siemens Healthcare Diagnostics GmbH) is used to measure blood glucose levels in routine clinical practice. This system measures blood glucose levels in fluoridated plasma by the hexokinase/glucose 6-phosphate dehydrogenase method. Glucose is phosphorylated to glucose 6-phosphate in the presence of ATP by hexokinase. Then, glucose 6-phosphate is dehydrogenated in the presence of NADP to 6-phosphogluconate by glucose 6-phosphate dehydrogenase. This reaction also yields NADPH2, which is measured by the system.

The hexokinase/glucose 6-phosphate dehydrogenase method is considered the reference method for assessing blood glucose levels, including in routine clinical practice. So far, no drugs are known to interfere with this measurement system [18].

All blood samples are to be collected from the site of insertion of the central venous catheter, as all blood samples will be collected from one lumen of the central venous catheter. Lithium or fluoride will be added to the blood samples and all blood samples will be processed immediately. The blood samples collected in lithium-heparinised tubes (Blutgas-Monovette®; Sarstedt AG & Co., Nümbrecht, Germany) used to assess agreement between the CGM and a BGA will be processed within 5 min. The blood samples collected in fluoride tubes (S-Monovette® Glukose; Sarstedt AG & Co.) used to independently confirm the results determined by a point-of-care BGA will be kept on ice until being sent to the central laboratory.

### Measurement process

All subjects will first enter a screening stage lasting up to 1 month, and an additional 2-h reference period before the study period. The study will consist of a 24-h measurement period and a 48-h follow-up period. During the measurement period, blood samples will be obtained 13 times. The overall duration of the measurement stage will be determined by the duration of intensive care.

### Screening (Visit 1)

All patients admitted to University Hospital Heidelberg will be assessed for eligibility after being fully informed of all aspects of the study that are relevant to their decision to participate. Patients will be asked to sign and date an informed consent form [19]. After providing consent, the subjects will undergo screening, which will involve assessment of baseline characteristics and medical history (Table 2).

**Table 2:** Study timetable

	**Visit 1**	**Visit 2**	**Visit 3 - 9**	**Visit 10**	**Visit 11 -12**	**Visit 13**	**Visit 14**	**Visit 15**
	up to one month and a reference period^1^ of 2 h before Visit 2	0 h^2^	1–7 h (every hour) ± 5 min	8 h ± 5 min	9 and 10 h ± 5 min	16 h ± 30 min	24 h ± 30 min	72 h ± 1 h
Informed consent	●							
Eligibility criteria	●	●						
Medical history	●							
Concomitant medication	●	●	●	●	●	●	●	●
Physical examination	●						●	
Vital signs^3^	●	●	●	●	●	●	●	●
Pregnancy test^4^	●							
Placement of the CGM		●						
CGM calibration		●						
Continuous glucose and lactate monitoring		●	●	●	●	●	●	up to every 2 h
Recalibration				●				
Central venous blood sampling for the BGA^5^			●	●	●	●	●	
Central venous blood sampling for central laboratory measurement^6^		●		●		●	●	
Removal of CGM								●
Assessment of unwanted events/effects		●	●	●	●	●	●	●

^1^ Priming, tempering and transient oscillation of the sensor systems.

^2^ Within 2 h of transfer to the recovery room or intensive care unit.

^3^ Heart rate, blood pressure, and tympanic body temperature.

^4^ Only females with reproductive potential.

^5^ Samples used to assess blood glucose, blood lactate, haematocrit, and haemoglobin.

^6^ Samples used to assess blood glucose and blood lactate levels.

CGM: continuous glucose monitor; BGA: blood gas analyser.

### Study period (Visits 2–14)

All subjects will be treated according to the local standards at the University Hospital Heidelberg. After successful induction of anaesthesia, a multi-lumen central venous catheter will be implanted in all patients. Perioperative antibiotic prophylaxis (e.g., 4 g mezlocillin and 500 mg metronidazole) will be administered approximately 30 min before commencing surgery, and will be repeated if the duration of surgery exceeds 4 h. Perioperative prophylaxis with unfractionated or fractionated/low-molecular-weight heparin will be administered to prevent venous thromboembolism. Patients will be closely monitored throughout surgery and the post-operative period.

**Figure 1:**
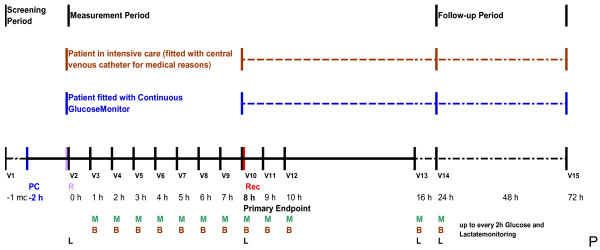
**Timeline.** PC: Priming and Calibration Period (Priming, tempering, transient oscillation and initial calibration of the sensor from the Continuous GlucoseMonitor); R: Referencing; Rec: Recalibration (duration = 10 min including washing); M: Glucose and Lactatemonitoring; B: Blood sampling for comparison (glucose, lactate, haemoglobin, haematocrit); L: Blood sampling for central lab (glucose and lactate).

Within 2 h after transfer to the recovery room or an intensive care unit, the patients will be re-assessed for eligibility and the planned measurements will be commenced, as summarised in the Figure 1 and Table 2. Unless medical reasons preclude entry into the study (i.e., a lumen is not required for medical purposes, such as perioperative fluid therapy and medications) the patients will be fitted with the CGM according to the manufacturer’s instructions. The device is to be primed, connected to the patient, and referenced to the blood glucose level measured by a BGA. This point represents the start of the measurement period (1 h before the first measurement).

For the next 10 h, the patients will be visited by the research staff every hour. At each visit, the CGM value will be recorded and blood samples will be collected for measurement by a BGA. At 0 and 8 h (Visits 2 and 10, respectively), additional blood samples will be collected and measured using the ADVIA 2400 Chemistry System to independently confirm the blood glucose measurement. Using the value obtained at 8 h (Visit 10), the CGM will be re-calibrated and further values will be recorded at 9, 10, 16, and 24 h. At 16 and 24 h (Visits 13 and 14, respectively), additional blood samples will be collected. Visit 14 represents the end of the measurement period. The CGM is left in place until Visit 15.

No subjects will be fitted with the CGM unless central venous cannulisation was necessary for medical reasons. The central venous catheters will be removed when clinically indicated. No subjects will be equipped or re-equipped with central venous catheters unless there is a clear medical reason. If discharge from the recovery room or intensive care unit occurs within 24 h, Visit 14 will be brought forward and the CGM will be removed.

### Follow-up period (Visit 15)

During the follow-up subjects will be visited at convenience (up to every 2 h). At each visit, the CGM values will be recorded and compared with values obtained as part of routine clinical practice.

### Notes

As the CGM values are available to the attending physicians responsible for perioperative patient care, these data might raise awareness of dysglycaemic events that might have been overlooked. The physicians can treat dysglycaemic events to the best of their knowledge and treatments should be initiated according to local standards once the CGM values have been confirmed by another device. The study protocol does not provide recommendations on how to treat dysglycaemic events.

Complications of central venous cannulisation have been recognised since the technique was first introduced into clinical use. Overall, > 15% of patients undergoing central venous cannulisation experience complications [20]. Although serious acute complications are rare when central venous cannulisation is performed by well-trained, experienced clinicians, infectious complications are relatively common, and the technique may result in significant morbidity and mortality. Considering these complications and their origin, all conceivable measures will be taken to prevent participants from any harm. Nevertheless, the study is not designed to investigate the efficacy or safety of central venous cannulisation. Therefore, central venous cannulisation is not considered a study-specific intervention.

### Statistical methods

The test statistic for testing the null hypothesis $H_{o}:\left|\delta \right|+2\sigma >\lambda$ is calculated as $Z=\frac{\left|\bar{d}\right|\pm 2s_{d}-\lambda }{\sqrt{3/ns_{d}^{2}}}$, where λ=20% for the outcome measure. Its distribution under $\left|\delta \right|+2\sigma =\lambda$ is asymptotically normal. Therefore, the null hypothesis is rejected if *Z* is below the 0.05 quantile of the normal distribution. Descriptive statistics of continuous variables and scores include the number of observations, the mean and standard deviation, the median, minimum, and maximum. Descriptive statistics of categorical variables (nominal or ordinal) include absolute and relative frequencies (n and per cent). The primary outcome measure is the level of agreement in blood glucose levels determined by the CGM and a BGA, assessed 8 h after the initial placement and calibration of the CGM using the relative difference $\bar{d}$, as described above. Bland and Altman plots will be drawn, including the CIs for the mean difference and limits of agreement to show the agreement between the pairs of values [9,21]. Secondary analyses, including the level of agreement between the two devices at each time after placement and calibration of the CGM will be analysed descriptively, while applying the same equivalence bounds used in the primary analysis. A linear mixed model will be developed using time as the fixed factor. Subject and the interaction subject × time will be included as random explanatory variables. The individual values $\bar{d}$ at all times up to 8 h will be included as the response variable. This model will be used to impute missing data. Biometric analysis will be defined in the statistical analysis plan, which must be authorised before the database is opened by the biostatistician, the sponsor, and the principal investigator.

## Discussion

Hyperglycaemia, insulin resistance [2,3], and metabolic acidosis often occur in critically ill patients. Several authors have independently, but consistently reported that intensive insulin therapy reduces morbidity in all patients and mortality in some patients [2,3,22-24]. Of particular interest in terms of the number of patients enrolled and methodological quality, are two trials conducted by Van Den Berghe et al. [2,3]. In 2001 Van Den Berghe et al. reported on a trial terminated after 1548 patients [2], as the fourth interim analysis revealed that conventional treatment (insulin infusion if the blood glucose level was > 215 mg/dL to maintain blood glucose levels between 180 and 200 mg/dL) was inferior to intensified insulin therapy (maintenance of blood glucose levels between 80 and 110 mg/dL). Morbidity and mortality were significantly reduced but intensified insulin therapy reduced morning blood glucose levels at the expense of a substantially greater risk of hypoglycaemia. In 2006, Van Den Berghe et al. reported on a subsequent trial enrolling 1200 patients [3]. Morbidity was significantly reduced but mortality was not. Again, intensive insulin therapy reduced morning blood glucose levels at the expense of a significantly greater risk of hypoglycaemia. However, results of the VISEP study at least partly conflicted with those of the studies by Van Den Berghe et al. The VISEP study was initiated to investigate the effects of intensive insulin therapy in a two-by-two factorial design [22]. The intensified insulin regimen was virtually identical to that described by Van Den Berghe et al. Blood samples used to adjust the continuous infusion of insulin were taken at least every 4 h. The trial was partially terminated for safety reasons after enrolling 488 patients. Intensive insulin therapy significantly reduced the mean morning blood glucose levels. However, this reduction did not result in any difference in outcome but did increase the risk of severe hypoglycaemia and the rate of serious adverse events. Considering these results, the devices currently in clinical use are not sufficient to detect impending hypoglycaemia [25]. Continuous assessment of blood glucose levels has several advantages. First, it shows the actual blood glucose levels at any time with no gaps between scheduled sampling and assessment times. Accordingly, it may detect hypoglycaemic and hyperglycaemic events that might have been missed. It may even help to predict these events in advance based on the trends in values. Therefore, the CGM would facilitate the use of intensive insulin therapy for seriously ill patients, and may improve the morbidity and mortality of these patients. the CGM may also be used within an artificial pancreas by forming a control loop, which consists of a tool for continuous measurement of blood glucose levels, an insulin pump, and an intravenous source of glucose for rescue [26]. However, no devices are currently available to continuously assess blood glucose levels in critically ill patients. Several attempts have been made to develop a device to continuously measure blood glucose levels within an intensive care setting [27,28] or to use the devices that were originally designed for diabetes management, as several of these devices are already available [25,29,30]. However, none of these devices were successful in intensive care settings. The results of this study and the introduction of the CGM may resolve this limitation by confirming the ability of the CGM to continuously measure blood glucose levels in intensive care settings.

### Study status

CONTASSGLU is part of the Verbundprojekt: Transfer eines Messverfahrens zur kontinuierlichen Glukosemessung in die medizinische Anwendung und deren präklinische und klinische Evaluierung, and is fully funded by the German Federal Ministry of Education and Research.

Approval for this study was provided by the Ethics Committee of the Medical Faculty Heidelberg (Mzmo-321/2011) and the applicable regulatory authorities (95.02 - 5660–6938) on 17 November 2011 and 1 February 2012, respectively. CONTASSGLU was registered at ClinicalTrials.gov on 17 April 2012 (NCT01580176). The study was initiated on 7 March 2012 and is intended to be open for enrolment for about 8 months.

## Abbreviations

BGA: RAPIDLab® 1265 blood gas analyser; CGM: Continuous GlucoseMonitor; CI: Confidence interval; GMA: German Medical Association.

## Competing interests

JS is Director of Engineering of Mechatronic AG. WK is Managing Director of TRACE Analytics GmbH. The other authors declare that they have no competing interests.

## Authors’ contributions

JBZ drafted the study protocol and the manuscript. ML helped to draft the study protocol and took over project management from JB. SH is conducting the study on site. JH helped to draft the study protocol and the manuscript, and is responsible for statistical analyses. CA supports SH. JW is helping to conduct the study on site. JS and WK helped to design the study. SL takes responsibility for the tasks performed by the Coordination Centre for Clinical Trials. JM is the principal investigator of the study, and participated in its design and realisation. MAW conceived the study, and participated in its design and coordination. All authors read and approved the final draft of the manuscript.

## Pre-publication history

The pre-publication history for this paper can be accessed here:

http://www.biomedcentral.com/1471-2253/12/24/prepub
